# Recent Therapeutic Approaches to Modulate the Hippo Pathway in Oncology and Regenerative Medicine

**DOI:** 10.3390/cells10102715

**Published:** 2021-10-11

**Authors:** Evan R. Barry, Vladimir Simov, Iris Valtingojer, Olivier Venier

**Affiliations:** 1 Merck & Co., Inc., Kenilworth, NJ 07033, USA; evan.barry@merck.com; 2 Sanofi, 94400 Vitry-sur-Seine, France; iris.valtingojer@sanofi.com; 3 Sanofi, 91380 Chilly-Mazarin, France

**Keywords:** Hippo pathway, YAP1/TAZ, TEAD transcription factors, palmitate pocket, TEAD binders

## Abstract

The Hippo pathway is an evolutionary conserved signaling network that regulates essential processes such as organ size, cell proliferation, migration, stemness and apoptosis. Alterations in this pathway are commonly found in solid tumors and can lead to hyperproliferation, resistance to chemotherapy, compensation for mKRAS and tumor immune evasion. As the terminal effectors of the Hippo pathway, the transcriptional coactivators YAP1/TAZ and the transcription factors TEAD1–4 present exciting opportunities to pharmacologically modulate the Hippo biology in cancer settings, inflammation and regenerative medicine. This review will provide an overview of the progress and current strategies to directly and indirectly target the YAP1/TAZ protein–protein interaction (PPI) with TEAD1–4 across multiple modalities, with focus on recent small molecules able to selectively bind to TEAD, block its autopalmitoylation and inhibit YAP1/TAZ–TEAD-dependent transcription in cancer.

## 1. Introduction

**Figure 1:**
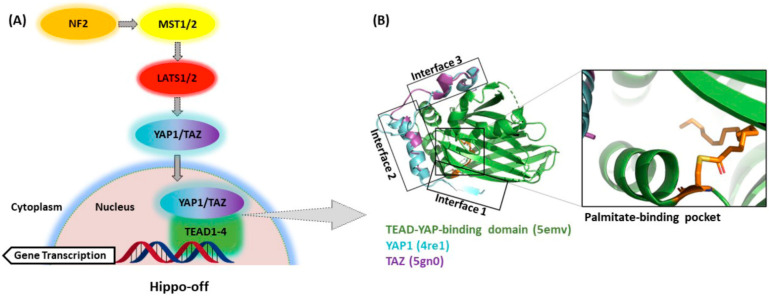
(**A**) In the Hippo-off state, unphosphorylated YAP1/TAZ accumulates in the nucleus and interacts with TEADs to drive the induction of genes involved in proliferation and cell survival. (**B**) Druggability assessment of the YAP1/TAZ–TEAD protein–protein interaction (PPI) has identified several druggable pockets amenable to small- and large-molecule inhibition, including interfaces 2 and 3, as well as the central lipophilic palmitate pocket (PDB: 5emv, 4re1 and 5gn0).

Originally described as an organ size and tissue growth control mechanism [1], the Hippo pathway has gained a tremendous amount of recognition in the fields of regenerative medicine and oncology in the past 16 years. Stunning organ growth phenotypes observed in the first mammalian liver models closely resembled those initially found in *Drosophila* studies, highlighting the conserved nature of this pathway, and its probable importance to human biology [2,3]. At a high level, Hippo functions as a tissue growth and cell proliferation inhibitory pathway. Extracellular stimuli activate the core kinase cascade that results in the phosphorylation of the transcriptional coactivators yes-associated protein 1 (YAP1) and transcriptional coactivator with PDZ-binding motif (TAZ, gene name *WWTR1*), resulting in their sequestration in the cytoplasm (Hippo-on state). When in the nucleus, YAP1 and TAZ interact with the TEAD transcription factors (TEAD1–4), the most well-known transcriptional mediators of the YAP1 and TAZ function (Hippo-off state) [4]. In the Hippo-off state (Figure 1A), the YAP1/TAZ–TEAD complex drives the induction of genes involved in proliferation and cell survival [5]. This interaction with TEADs is critical for the growth-promoting properties of YAP1 and TAZ as many of the effects of the YAP1 transcriptional activity can be blunted by eliminating the YAP1–TEAD interaction [6,7,8,9]. Many of the growth-promoting phenotypes observed after YAP1 activation eventually lead to tumor formation, and thus TEADs likely play an integral role in this process [3,8,10,11,12]. Whether the four TEAD protein family members, TEAD1–4, have the same or slightly different functions in this process is not yet understood and still a matter of research (reviewed in [13]. From the structure and sequence points of view, all TEAD proteins share the same domain structures and are highly homologous in their YAP1-binding domain [14]. 

Based on the strong growth-promoting and tumor-initiating phenotypes in mice and *Drosophila*, it is unsurprising that the Hippo pathway would play an integral role in human neoplasms. Indeed, overexpression of, or increased levels of nuclear localized YAP1 have been found in numerous solid tumor indications. In addition to general overexpression or accumulation of YAP1 staining in tumor cells (often termed “YAP1 activation”), several members of the Hippo pathway are known to be altered in human cancer at the DNA level, including amplification of *YAP1* and *TAZ,* as well as genomic deletions or truncating mutations in *NF2, LATS1* and *LATS2* [15,16]. Although much of the early research related to the oncogenic potential of YAP1 and TAZ centered on their role as cell-autonomous drivers, there is growing evidence that these transcriptional cofactors might play a much wider function than simply stimulating proliferation or inducing antiapoptotic factors. It is becoming clear that YAP1 and TAZ are multifaceted regulators of the processes driving tumor growth. Some of these attributes include, but are not limited to, (1) broadly regulating the tumor microenvironment (TME), including impacting antitumor immunity, (2) driving resistance to a wide array of drugs (including cytotoxic and targeted agents) and (3) acting as a tumor-intrinsic oncogenic driver [4,17,18]. Until very recently, it has been difficult to systematically probe the tumor growth requirements for the YAP1/TAZ–TEAD interaction on a broad scale. Additionally, many published studies looking at the role of YAP1/TAZ and TEAD in regulating tumor growth rely on analyzing tumor initiation. Whether YAP1/TAZ and TEADs are critical for the continued growth of already established tumors is still a largely unanswered question critical to successfully targeting YAP1/TAZ–TEAD in cancer.

Generally considered to be extremely difficult or impossible to drug by traditional small-molecule approaches, transcription factor/cofactor complexes are of critical importance to cellular differentiation, as well as diseases, including cancer, and thus represent highly attractive drug targets [19]. Although YAP1 and TAZ lack a defined druggable pocket, this is not the case for TEAD proteins, which harbor a recently uncovered hydrophobic pocket (Figure 1B) containing a cysteine residue conserved in each of the four TEAD family members residing in the YAP1-binding domain [20,21,22]. This conserved cysteine is the site of *S*-palmitoylation and is thought to modulate TEAD stability and, thus, transcriptional output [18]. This newly discovered foothold for medicinal chemistry efforts could pave the way to selective and on-target TEAD allosteric inhibitors that inhibit palmitoylation and thus disrupt YAP1/TAZ–TEAD transcriptional activity. In addition to the allosteric palmitate pocket, TEAD interfaces 2 and 3 have also been identified as druggable pockets within the YAP1-binding domain (YBD), albeit more amenable to the binding of larger molecules (e.g., peptides, peptidomimetics, etc.) able to directly disrupt the YAP1/TAZ–TEAD protein–protein interaction (PPI). Many questions surrounding the utility and feasibility of targeting this transcription factor complex still persist, however. In this review, we discuss some of the potential oncology settings where a drug-targeting YAP1/TAZ–TEAD would be of interest and what progress has been made towards the discovery of novel, selective and high-quality inhibitors of this critical transcription factor/cofactor complex. We highlight several molecules that have now either entered phase I clinical trials or are slated to do so in the near future.

## 2. Opportunities to Modulate the Hippo Pathway

### 2.1. YAP1 in Embryogenesis and Developed Tissues

In a healthy organism, YAP1 activation is apparent during embryonic development where cell proliferation and tissue growth are essential. Homozygous *Yap1*-knockout mouse embryos arrest development around embryonic day 8.5 and have defects in yolk sac vasculogenesis and embryonic axis elongation [23]. Similarly, conditional deletion of *Yap1* in the embryonic heart or lung impair cardiomyocyte proliferation and the development of basal airway stem cells in the lungs, respectively [24]. Conversely, in developed adult tissues, YAP1 activity is kept at a low steady-state level through the continuous and active signaling control from the upstream Hippo pathway. Induction of YAP1 in adults is restricted to the temporary activation in response to injury, where YAP1 initiates short-term transcriptional programs required for wound healing and repair processes (reviewed in [25]). Thus, the conditional knockout of *Yap1* in adult mouse intestines had no consequences for the normal intestinal function but did impair tissue repair post-dextran sodium sulfate (DSS) injury [26]. Indeed, the therapeutic activation of YAP1 has been considered for the regeneration of injured organs, a subject covered in more depth in other reviews [25,27,28].

### 2.2. YAP1 as a Tumor-Intrinsic Oncogenic Driver

**Figure 2:**
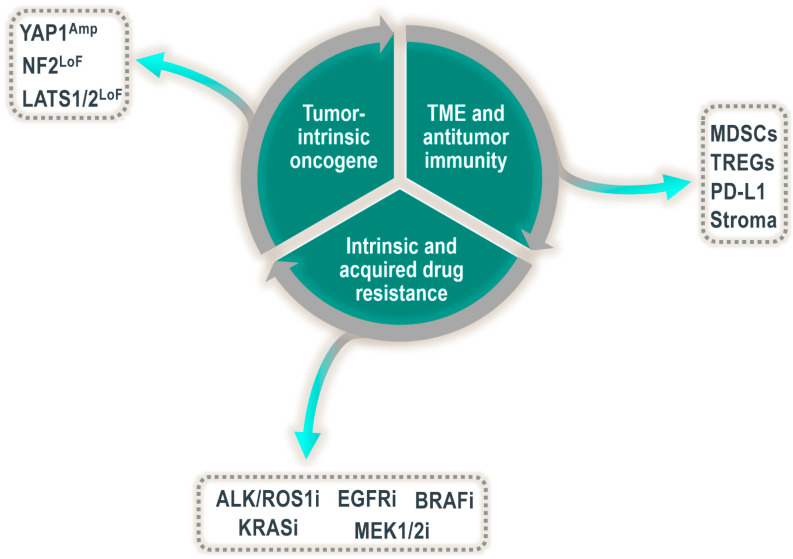
Multifaceted role of YAP1/TEAD in driving tumor growth and/or progression. Amp = amplification; LoF = loss of function; ALK/ROS1i, EGFRi, BRAFi, KRASi, MEK1/2i = inhibitors of ALK/ROS, EGFR, BRAF, KRAS and MEK.

In contrast to the temporary activation of YAP1 observed in wound healing, the continuous and persistent activation of YAP1 in adult tissues is associated with cancer and resistance to therapy (Figure 2). Many different cancer types harbor aberrant YAP1 activation, and examples range from tumors of the lungs, liver, skin and pancreas to tumors of the breasts, uterus, prostate, head and neck cancers and gliomas (reviewed in [17,29]). However, in which cases YAP1 is a functional driver in these malignancies still requires further investigation. In some of the abovementioned indications, the origin of the YAP1 activation can be directly traced to genetic alterations in the Hippo pathway or in YAP1 itself. For example, analysis of the TCGA database of patient tumors demonstrates that a subset of roughly ten percent of patients with cervical cancer shows focal amplification of *YAP1* [30], which in turn is associated with an increase in YAP1 transcriptional activity. Similarly, a large set of more than sixty percent of patients with malignant pleural mesothelioma, an asbestos-induced tumor of the lungs, harbor genetic alterations in the Hippo signaling pathway or its direct upstream regulators. These alterations predominantly comprise deletions or loss-of-function mutations in *LATS1* or *NF2* and are, once again, linked to an increase in the transcriptional activity of YAP1 [30].

For malignant mesothelioma tumors, it has been shown that the aberrant activation of YAP1 is an actual driver of tumor maintenance. The inhibition of YAP1 in preclinical models of malignant mesothelioma with Hippo pathway alterations decreased YAP1-dependent gene transcription and inhibited tumor cell growth. This effect was initially demonstrated by two small-molecule inhibitors of TEAD palmitoylation, as well as by genetic studies. These observations were found both in vitro as well as in vivo [31,32,33]. Specifically, the inhibition of YAP1 activation and the subsequent reduction of already established tumors in vivo clearly demonstrate that YAP1 is a driver of tumor maintenance in malignant mesothelioma with the Hippo pathway or *NF2* deletions. Furthermore, *NF2* deletions are also found in other tumor types such as renal cell carcinoma, cervical squamous cell carcinoma, schwannomas and meningiomas, which may hence respond equally well to the inhibition of YAP1 activation [33,34].

### 2.3. YAP1 as a Mechanism of Intrinsic and Acquired Drug Resistance

Not all cancers with YAP1 activation are responsive to the inhibition of YAP1 alone. In many contexts, YAP1 is not the driver of tumor growth but rather an acquired mechanism that adds to a set of already present driver alterations and increases tumor aggressiveness or resistance to therapy (Figure 2). In lung cancer, for example, YAP1 activation confers resistance to therapies targeted against common lung cancer oncogenes, such as anaplastic lymphoma kinase (ALK), BRAF or mutated epidermal growth factor receptor (EGFR) tyrosine kinase. The mechanism of action by which YAP1 confers resistance to EGFR-targeted therapies is best understood and seems to be linked to the concept of persister cells, a small set of cells that resist treatment to therapy. In EGFR mutant non-small-cell lung cancer (NSCLC), it has been shown that the population of persister cells remaining viable after the combined treatment with the EGFR and MEK inhibitors osimertinib and trametinib, respectively, is in fact YAP1-activated and permits tumor outgrowth upon treatment stop. In turn, genetic downregulation of YAP1 resensitizes these tumors to osimertinib and trametinib treatment [35,36]. Mechanistically, Kurppa et al. attribute the role of YAP1 in this context to the evasion of apoptosis by YAP1–TEAD-mediated repression of the proapoptotic protein BMF via the EMT transcription factor SLUG. YAP1 activation itself in response to osimertinib or trametinib treatment is thought to be the result of epigenetic changes. Along the same lines, YAP1 activation was associated with poor response to treatment with the ALK/ROS1 inhibitor crizotinib in ALK-rearranged lung cancers. *YAP1* silencing suppressed tumor growth in resistant cells, patient-derived xenografts, and *EML4–ALK* transgenic mice, whereas YAP1 overexpression decreased the responsiveness of parental cells to the ALK inhibitor [37]. Similarly, YAP1 activation is a mechanism of resistance to crizotinib therapy for ROS1-rearranged lung cancers [38].

YAP1 activation is also a major resistance mechanism in other tumor indications with increased MAPK pathway signaling. Treatment of BRAF mutant skin tumors with BRAF or MEK inhibitors vemurafenib and trametinib leads to resistance to therapy through YAP1 activation via actin cytoskeleton remodeling and RhoA-mediated inhibition of Hippo kinase LATS1 [39,40]. A recent cellular screen identified YAP1 activation as a novel mechanism of resistance to the triple combination of EGFR, BRAF and MEK inhibitors cetuximab, dabrafenib and trametinib in BRAF mutant colorectal cancer cell lines [41], which is in line with studies showing that the knockdown of YAP1 increases sensitivity to mitogen-activated protein kinase (MAPK) pathway inhibitors [42].

Finally, YAP1 activation is also described as a bypass mechanism to KRAS inactivation in cancers driven by oncogenic mutant KRAS signaling. Most of these studies are based on genetic knockdown or knockout of KRAS since specific inhibitors of mutant KRAS proteins are not yet available for most KRAS mutant forms. Thus, *Yap1* gene amplification appears to occur in mice under KRAS ablation in a KRAS G12D-dependent mouse pancreatic tumor model [7], while a genome-scale cDNA screen identified activation of YAP1 as a factor for survival upon KRAS knockdown in KRAS G13D mutant colorectal cancer cell lines [43]. YAP1 upregulation was further identified as a mediator of resistance to combined TBK1 and MEK inhibition in KRAS mutant lung cancer cells and in a KRAS G12D mutant genetically engineered lung cancer mouse models [44]. The mechanisms by which YAP1 activation bypass KRAS inactivation span from (1) the induction of the epithelial mesenchymal transition (EMT) through (2) the RAS–YAP1 mediated activation of transcription factor FOS to (3) the RAS-mediated protein stabilization of YAP1 via the downregulation of SOCS-box proteins which serve as substrate recognition modules of elongin B/C ubiquitin ligase complexes [45] or (4) the stabilization of the YAP1-binding partners TEADs [46]. Studies in *Drosophila* suggest that Hippo–YAP1 signaling controls the transcriptional response to RAS via the transcriptional regulators Pointed and Capicua [47].

While YAP1 activation is a major bypass mechanism to EGFR and MAPK pathway inhibition, the role of YAP1 as a mechanism of resistance to therapy seems to be general in many cancer types and independent of the type of treatment used. Numerous reports describe the activation of YAP1 in response to chemotherapy treatment covering resistance to anti-microtubule, antimetabolite and DNA-damaging agents [48]. In this context, YAP1-mediated regulation of ferroptosis is starting to be discussed as one of the potential mechanistic links between chemoresistance and high YAP1 activity [49,50,51,52,53]. In addition, an increasing number of studies have shown that the Hippo pathway is closely linked to endocrine therapy resistance in breast and prostate cancer [54,55].

In summary, YAP1-mediated resistance to therapy typically includes an initial upregulation of YAP1 activity. This upregulation may result from the inactivation of the NF2–Hippo axis via mechanisms such as genetic alterations (for example, of the *NF2* and *LATS1/2* genes), inhibition of LATS kinases through RhoA signaling or downregulation of *LATS* / upregulation of *YAP1* mRNA via micro RNAs [56,57]. Additionally, YAP1 activation may come from signals increasing YAP1 gene expression, protein half-life or nuclear translocation. The consequence of YAP1 activation is always an increased transcriptional output of YAP1 target genes leading to resistance to therapy by evasion of apoptosis (e.g., through upregulation of apoptosis inhibitors CYR61, CTGF and antiapoptotic BCLXL), increase of cell proliferation (e.g., through upregulation of growth factors such as EGFR, HER3 and AXL) or EMT transition (e.g., through interaction with transcription factors SLUG and FOS). Whether all these mechanisms for YAP1 activation and subsequent resistance to therapy exist in parallel and within the same tumor type or tumor cell requires further investigation. It is also important to better understand under which of the circumstances YAP1 requires partnering with TEAD, as this will impact the potential to pharmacologically target resistance to therapy.

### 2.4. Regulation of Immunity and the Tumor Microenvironment by YAP1/TAZ

The tumor microenvironment (TME) is a multifaceted ecosystem composed of a myriad of cell types and resources, each critical to the growth and survival of the tumor as a whole. Indeed, tumors undergo evolutionary processes during development driven by the tumor cells and the influence of the surrounding microenvironment [58]. The preponderance of published literature suggests that YAP1 and TAZ are critical in the evolution of some tumors, in part via their influence on the TME (Figure 2). The tumor immune microenvironment is a key component of the TME and is a critical regulator of neoplastic growth. YAP1 and TAZ are likely involved in this facet of tumor biology as intrinsic regulators of certain immune cell subsets as well as extrinsic regulators of the immune response via their oncogenic driver role in tumor cells [17,59,60].

Although YAP1 and TAZ are not required for normal hematopoiesis [61], they may be important for the function of some subsets of immune cells, as several groups have shown that YAP1 and TAZ are critical regulators of specialized T cells in a context-specific manner [62,63,64,65,66]. The function of TAZ in T cells has mainly been described as a regulator of the balance between Th17 and Treg differentiation, likely with implications for regulating autoimmunity [65]. Loss of TAZ results in CD4+ T cell differentiation to skew towards immunosuppressive Tregs at the expense of the proinflammatory Th17 subset. Knockout (KO) of YAP1 in naïve T cells results in hypersensitivity to in vitro stimulation and enhanced antitumor activity in vivo [62,63,64,66]. Interestingly, YAP1 KO CD4 and CD8+ T cells demonstrated superior tumor infiltration compared to wild-type counterparts, and tumor growth was either greatly delayed or nearly absent in tumor-bearing CD4–Cre or Foxp3–Cre mice [62,63,64]. These observations suggest that inhibition of YAP1 activity could help drive more profound antitumor T cell responses. YAP1 appears to overall play a functionally suppressive role in T cells; however, it is currently unclear if the interaction with TEAD is critical in this process [66], which has important implications for directly drugging YAP1-driven processes with small-molecule approaches. It is possible that YAP1 plays a role in the cytoplasm of T cells, modulating the activity of NFAT1 [66], suggesting that treatment modalities that force cytoplasmic localization of YAP1 could be of interest, for example, statins or SRC inhibitors (e.g., dasatinib) [67]. In preclinical mouse models, dasatinib has been shown to improve the antitumor activity of anti-PD1 antibodies, and prior statin use may be associated with improved patient outcomes in non-small-cell lung cancer as well as in mesotheliomas, although further studies are required to confirm this observation [68,69,70]. It will be interesting to see clinically how YAP1 and TEAD factor into these observations.

Both YAP1 and TAZ have also been shown to regulate the tumor immune microenvironment via numerous avenues, including tumor cell-intrinsic properties via directly regulating the expression of PD-L1, as well as recruitment of immunosuppressive cell types via induction of secreted factors [71,72,73,74,75,76,77]. YAP1 has also been shown to promote adaptive resistance to anti-PD1 therapy [78], and the YAP1/TAZ target gene signature has been shown to correlate with tumor immune cell infiltration in patient samples [16]. Direct upregulation of PD-L1 transcripts in tumor cells by both YAP1 and TAZ has been shown in multiple contexts and involves their interaction with TEAD transcription factors [72,73,74,75]. These observations are potentially of interest since tumor cells upregulate PD-L1 to avoid immune surveillance. Therefore, inhibitors of YAP1/TAZ could presumably alleviate some of this immune escape; however, testing this mechanistically in an immunocompetent setting may be challenging if YAP1/TAZ do not directly regulate PD-L1 in mouse cells [73]. Other mechanisms of immunosuppression have been linked to secreted factors induced by YAP1. These include (1) tumor stromal cell expansion via the expression of CTGF, CYR61, IL6 and MMP7 [76], (2) macrophage polarization [71] in pancreatic cancer models and (3) CXCL5-mediated expansion of myeloid-derived suppressor cells in prostate cancer [77]. It is clear from the current literature that the transcriptional effectors of the Hippo pathway likely drive the immunosuppressive tumor microenvironment on numerous fronts and it appears to be an attractive target from the antitumor immunity perspective.

## 3. Targeting the YAP1/TAZ–TEAD Interaction

**Figure 3:**
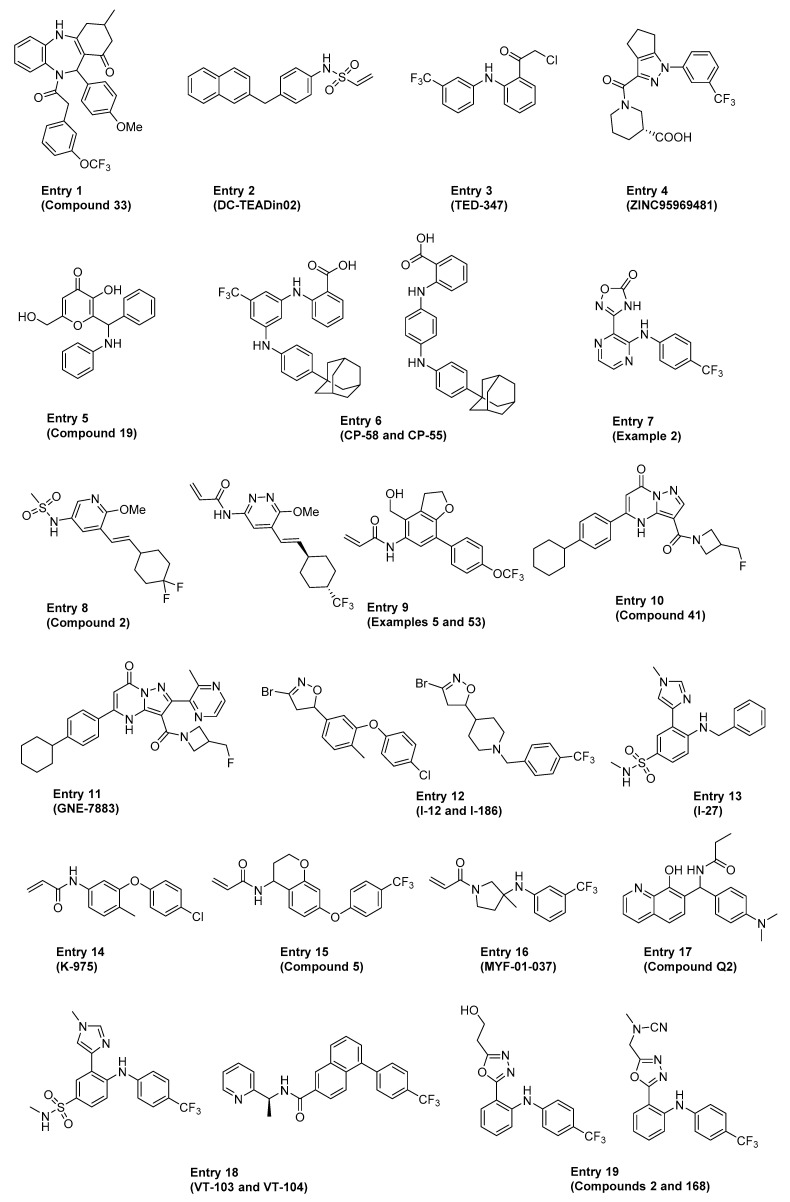

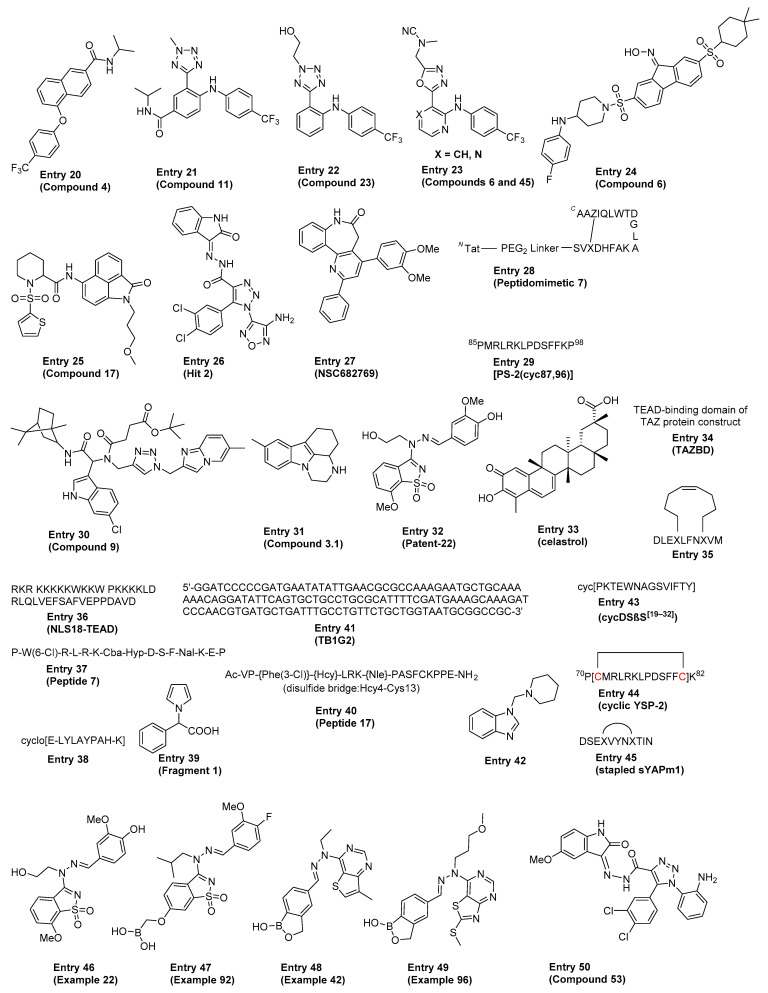
Structures of representative compounds with claims to disrupt the YAP1–TEAD interaction.

Several excellent reviews covered the various direct and indirect approaches to targeting the Hippo pathway in cancers and regenerative medicine, including a detailed discussion on the historical proof-of-concept tool molecules such as verteporfin, flufenamic acid, Peptide 17 and Super-TDU [79,80,81,82,83]. The ensuing section highlights some of the more recent advancements in the field, focusing on compounds and therapeutic approaches directed at disrupting the YAP1/TAZ–TEAD interaction either directly (i.e., classical PPI inhibition, also referred to as just “PPI” in Table 1) or allosterically by blocking TEAD palmitoylation and stabilization (referred to as “Allosteric, palmitate” in Table 1). The comprehensive summary in Table 1 is meant to provide the reader with an overview of the existing chemical matter shown in Figure 3 that spans across multiple modalities to disrupt the YAP1/TAZ–TEAD interaction. In cases where patent publications are discussed, we decided to highlight representative examples based on the data disclosed.

As previously discussed, YAP1/TAZ binding to TEAD regulates the transcriptional output of the Hippo pathway and therefore represents a promising strategy to target diseases with dysregulated Hippo signaling. YAP1 and TAZ are natively disordered proteins and therefore difficult to selectively target with traditional small-molecule or peptide modalities [84]. Instead, recent therapeutic approaches have focused on targeting the TEAD proteins, which consist of two well-defined DNA-binding and YAP1-binding domains. Targeting the DNA-binding domain of TEAD (DNA-TEAD) represents a viable and attractive strategy to regulate TEAD-mediated gene expression; however, this approach has yet to gain momentum and warrants additional attention [85]. Alternatively, recent efforts within the Hippo field have focused on targeting the TEAD coactivator domain (YAP1/TAZ–TEAD, also referred to as YAP1–TEAD below). The domain architecture and structure of YAP1/TAZ and TEAD have been extensively reviewed before, with the availability of several crystal structures of TEADs complexed with either a coactivator or small-molecule partner significantly enhancing our understanding of how to disrupt the YAP1–TEAD interaction [85,86]. These reviews discuss progress towards targeting the most druggable sites within the YAP1–TEAD binding domain (YBD), initially focusing on interfaces 2 and 3 shown in Figure 1B [4,85,86,87,88,89,90]. These early approaches to directly targeting the YAP1–TEAD PPI have primarily relied on larger molecules and peptidomimetics designed to bind to the YBD of TEAD and therefore directly inhibit formation of the transcriptionally active YAP1–TEAD complex. However, a recent development in targeting the Hippo pathway has been the discovery of a central lipophilic pocket in TEAD amenable to small-molecule binding that is the site of autopalmitoylation [20,22,91]. Within this lipophilic (palmitate) pocket, posttranslational *S*-palmitoylation of TEAD at a conserved catalytic cysteine (Cys) residue (e.g., C380 in TEAD2) leads to TEAD stabilization [22] and is believed to be critical for maintaining appropriate protein folding to enable formation of the transcriptionally active YAP1–TEAD complex. Therefore, targeting the palmitate pocket with small molecules to allosterically inhibit and disrupt formation of the YAP1–TEAD complex represents an attractive strategy to modulate TEAD-driven gene transcription. Within the palmitate pocket, it is important to note that the sequence similarity is 80–90% across the four TEAD isoforms, suggesting that the discovery of pan-TEAD ligands is feasible [22]. However, key questions in targeting TEAD palmitoylation remain with respect to the impact of isoform selectivity on both efficacy and depth of response, as well as safety.

**Table 1:** Reported key in vitro and in vivo data for each representative example in Figure 3 (MOA = mechanism of action; NR = not reported).

Entry (Ref.)	MOA	In Vitro Data	In Vivo Data
1 [92]	NR	A549-CTGF_Luc = 200 nM Dose-dependent decrease of CTGF and YAP1/TAZ mRNA in 293T and MDA-MB-231 cells	NR
		Antiproliferation activity in 20 tumor cell lines with IC_50_ < 10 µM (e.g., SMMC-7721 IC_50_ = 3.1 µM)	
2 [93]	Allosteric, palmitate (covalent)	TEAD auto-palmitoylation inhibitor IC_50_ = 197 nM	
		Downregulation of TEAD-specific genes (CYR61 and CTGF) by RT-PCR	NR
3 [94,95]	Allosteric, palmitate (covalent)	Inhibition of patient-derived spheroids of GBM43 cell lines (30% at 10 µM)	NR
		Dose-dependent downregulation of CTGF by RT-PCR	
4 [96]	Allosteric, palmitate	NR (lead molecule identified as a more potent inhibitor of TEAD2 than flufenamic acid by molecular dynamics and ADMET predictions)	NR
5 [97]	Allosteric, palmitate (covalent)	Inhibition of the TEAD–YAP1 interaction by FP-based competitive binding assay using FITC-labeled hYAP_50–100_ at IC_50_ = 70 nM Target engagement in HCT116 by CETSA (hTEAD4 ΔTm = 3.3 °C) 90% reduction of ANKRD1 mRNA levels (TEAD target gene) at 6 h in HEK293	NR
6 [98]	Allosteric, palmitate	92.1 cell proliferation: IC_50_ of CP-58 = 5.087 µM, of CP-55 = 0.03821 µM Huh7 cell proliferation: IC_50_ of CP-58 = 4.865 µM, of CP-55 = 0.3289 µM	NR
7 [99]	Allosteric, palmitate	MCF7 TEAD RGA IC_50_ = 41 nM Thermal shift assay hTEAD2 ΔTm = 10.6 °C	NCI-H226 xenograft efficacy study: ~100% TGI at 250 mg/kg PO (62 days)
8 [100,101]	Allosteric, palmitate	Lipid FP assay IC_50_ = 182 nM (TEAD1), 603 nM (TEAD2), 396 nM (TEAD3) and 158 nM (TEAD4) TEAD2 PPI TR-FRET assay IC_50_ = inactive Detroit X1 562 cell reporter assay IC_50_ = 31.8 nM Dose-dependent downregulation of mRNA expression of CYR61 and CTGF in HUH-7, JHH-7, MDA-MB-231 and Detroit X1 562	Detroit X1 562 xenograft efficacy study: %TGI (lower, upper) is 75% (52, 89) with ABT + 150 mg/kg Compound 2 and 78% (55, 92) with 200 mg/kg Compound 2
9 [102]	Allosteric, palmitate (covalent)	Lipid HTRF IC_50_ for Example 5 (TEAD1, TEAD2, TEAD3, TEAD4) = 0.03, 0.02, 0.07, 0.01 µM Lipid + 4 h HTRF IC_50_ for Example 53 (TEAD2, TEAD4) = 0.0034, 0.00355 µM	NR
10 [103]	Allosteric, palmitate	HTRF TEAD–YAP_50–100_ disruption assay EC_50_ = 0.0045 µM (TEAD2), 0.01 µM (TEAD3)	NR
11 [104]	Allosteric, palmitate	Lipid HTRF IC_50_ (TEAD1, TEAD2, TEAD3, TEAD4) = 34, 14, 37, 13 nM YAP1 HTRF IC_50_ (TEAD1, TEAD2, TEAD3, TEAD4) = 39, 13, 93, 34 nM Cell proliferation EC_50_ (OVCAR-8, NCI-226) = 115, 333 nM	NCI-H226 xenograft study: 102% TGI at 250 mg/kg SC of GNE-7883
12 [105]	Allosteric, palmitate (covalent)	MCF7 TEAD cell reporter assay EC_50_ for I-186 H226 cell proliferation assay EC_50_ for I-186 < 100 nM H28 cell proliferation assay EC_50_ for I-186 > 500 nM Cell proliferation assay EC_50_ for I-12 (Isomer 2) < 200 nM for MSTO211H, H226, H1975, H2052, H2085, SNU182, U251, YD8, but inactive in H28	NCI-H226 tumor xenograft efficacy study with I-12 (Isomer 2): 82.6% TGI at 50 mg/kg IP/QD NCI-H226 xenograft study with I-186 (Isomer 1): 67.3% TGI at 75 mg/kg PO/QD Comparable efficacy in the MSTO211H tumor xenograft efficacy study shown with I-186
13 [106]	Allosteric, palmitate	MCF7 TEAD cell reporter assay EC_50_ < 100 nM H226 cell proliferation assay EC_50_ < 100 nM H28 cell proliferation assay EC_50_ > 500 nM	NCI-H226 PD model: > 50% downregulation of CTGF mRNA in the tumor when dosed with I-27 at 30 mg/kg PO
14 [32]	Allosteric, palmitate (covalent)	Dose-dependent PPI disruption H226 of the YAP1–TEAD1/4 PPI and TAZ–TEAD1/4 by Co-IP in H226 K-975 inhibited cell proliferation more potently in NF2-non-expressing cell lines as part of a 14 cell line mesothelioma panel	NCI-H226 tumor xenograft efficacy study: tumor stasis at 100 mg/kg PO and tumor regression at 300 mg/kg PO MSTO-211H tumor xenograft efficacy study: tumor stasis at 300 mg/kg PO
15 [107]	Allosteric, palmitate (covalent)	GI_50_ < 100 nmol/L against the human mesothelioma cell line, NCI-H226	NR
16 [35,108]	Allosteric, palmitate (covalent)	100% inhibition of TEAD-driven transcription (8XGTIC) Pretreating cells with MYF-01-37 led to the loss of direct TEAD pulldown by biotin–MYF-01-037 Inhibition of the direct YAP1–TEAD interaction in HEK293T cells (IC_50_ = 0.8 µM) leads to the reduction in CTGF expression in PC-9 cells, which can be overturned by the overexpression of a TEAD1 C359S mutant (IC_50_ = 8.1 µM)	NR
17 [109]	Allosteric, palmitate	TEAD binding by SPR with K_d_ = 2.6 µM Dose-dependent increase in the TEAD dual luciferase reporter activity in HEK293 (EC_50_ = 2.6 µM) Increase in endogenous expression levels of TEAD target genes CTGF, CYR61 and ANKRD1 by RT-PCR	Quinolinol Q2 accelerates cutaneous wound healing in mice, which was already notable at day seven
18 [33,110,111]	Allosteric, palmitate	Thermal shift assay for VT-103 ΔTm: 8.3 °C (TEAD1), 4.1 °C (TEAD2), 1.0 °C (TEAD3), 1.9 °C (TEAD4) Thermal shift assay for VT-104 ΔTm: 8.6 °C (TEAD1), 5.4 °C (TEAD2), 8.2 °C (TEAD3), 4.3 °C (TEAD4) Cell proliferation inhibition IC_50_ for VT-103: 7.13 nM (H2052), 15.2 nM (H2373), 3.8 nM (H226), > 3 µM (H28, H2452 and MSTO-211H) Cell Proliferation Inhibition IC_50_ for VT-104: 31.6 nM (H2052), 25.6 nM (H2373), 16.1 nM (H226), > 3 µM (H28, H2452 and MSTO-211H)	NCI-H226 tumor xenograft efficacy study with VT-103: TGI = 106.14% at 3 mg/kg PO QD NCI-H226 tumor xenograft efficacy study with VT-104: TGI = 102.49% at 3 mg/kg PO QD NCI-H2373–Tu–P2 tumor xenograft efficacy study with VT-103: TGI = 126.70% at 10 mg/kg PO QD
19 [112]	Allosteric, palmitate	HEK293T YAP1 reporter assay IC_50_ < 100 nM	NR
20 [113]	Allosteric, palmitate	HEK293T YAP1 reporter assay IC_50_ < 100 nM	NR
21 [114]	Allosteric, palmitate	HEK293T YAP1 reporter assay IC_50_ < 100 nM	NR
22 [115]	Allosteric, palmitate	HEK293T YAP1 reporter assay IC_50_ < 100 nM	NR
23 [116]	Allosteric, palmitate	HEK293T YAP1 reporter assay IC_50_ < 100 nM	NR
24 [117]	NR	HEK293T YAP1 reporter assay IC_50_ < 100 nM	NR
25 [118]	NR	HEK293T YAP1/TEAD luciferase reporter assay: 78% inhibition Cell proliferation in HT29 EC_50_ = 6.91 µM	Tumor size growth was decreased in an AOM/DSS orthotopic syngenic mouse model using 50 mg/kg IP QD dosing of Compound 17. No effect on body weight and decrease in % of Treg cells were also noted FACS analysis confirmed that Treg cells decreased
26 [119]	PPI	HEK293T TEAD luciferase reporter assay IC_50_ = 6.5 µM Decrease in the relative mRNA expression of AXL, CYR61 and CTGF measured in MDA-MB-231	NR
27 [120]	PPI	NSC682769 binds YAP1 in a concentration-dependent manner with K_d_ of 738 nM by SPR Strong correlation between IC_50_ and the relative nuclear YAP1 expression (R^2^ = 0.8267) in a proliferation panel of seven GMB cell lines	83% TGI in SCID mice implanted with subcutaneous LN229 xenografts with 20 mg/kg NSC682769. Overall survival increased from 26 days (control) to 70 days (20 mg/kg NSC682769)
28 [121]	PPI	Proteomimetic 7 (Tat–PEG_2_–4E) stimulates the expression of TEAD target genes CYR61, CTGF, ANKRD1 and SPINE in human cardiomyocytes Increase in YAP1 nuclear translocation and cell cycle activity in primary juvenile rat heart cells, which is required for cardiomyocyte proliferation. Cell cycle activity stimulated to the same extent as positive controls SB203580 (p38MAPK inhibitor) and CHIR99021 (GSK3β inhibitor)	NR
29 [122]	PPI	Constrained by adding a disulfide bond across the spatially vicinal residue pair Arg87–Phe96 at the peptide’s two ends, PS-2(cyc87,96) exhibits affinity towards TEAD4 K_d_ = 21 μM	NR
30 [123]	Allosteric, palmitate	Human TEAD4 binding confirmation by nanoDSF with ΔT_m_ = 3.1 °C Inhibition of palmitic acid binding to the hTEAD4 central pocket measured by fluorescence polarization with IC_50_ = 0.41 μM Inhibition of YAP1 binding to hTEAD4 measured by fluorescence polarization with IC_50_ = 6.75 μM Dose-dependent downregulation of CTGF mRNA levels in HEK293 upon Hippo signaling inhibition by XMU–MP-1	NR
31 [124]	PPI	HeLa Gal4–NLUC IC_50_ (TEAD1, TEAD2, TEAD3, TEAD4) = 40 μM, 33 μM, 44 μM, 36 μM Dose-dependent inhibition of HeLa cell proliferation at doses > 40 μM Dose-dependent inhibition of RaVSMC and human VSMC cell proliferation with EC_50_ of 10 μM and 1.5 μM, respectively	NR
32 [125]	PPI	FP competition assay in the presence of palmitoylated Pal–TEAD2 to confirm YAP1–TEAD PPI disruption at interface 3 with IC_50_ = 740 μM	NR
33 [126]	PPI	Celastrol inhibits the SmBiT–YAP1/LgBiT–TEAD interaction in a dose-dependent manner and inhibits YAP1/TAZ–TEAD biosensor activities in vitro and in vivo Celastrol significantly inhibited cell proliferation and decreased cell viability in H1299 lung and MDA–MB-231 breast cancer cells. It also significantly inhibited cell growth in H1299 (up to 88% reduction at 5 µM)	NR
34 [127]	PPI	Co-IP assay showed that TEAD can be successively coimmunoprecipitated with TAZ in U251 and U87 cells >50% reduction in the number of U251 and U87 colonies transfected with TAZBD relative to the control vector	Mice injected with U251, U251 vector or U251–TAZBD harvested cells were monitored for the tumor growth rate. TAZBD-expressing cells showed a slower rate of tumor development relative to the control vector
35 [128]	PPI	Stapled TAZ-derived α-helical peptide showed K_d_ affinity in fluorescence polarization assay of TEAD1 = 67.5 µM, TEAD2 = 274 µM, TEAD3 = 9.8 µM, TEAD4 = 29.4 µM	NR
36 [129]	PPI	NLS18–TEAD-induced apoptosis of MDA-MB-231 cells (63% at 25 µM)	NLS18–TEAD exhibits the antitumor effect (TGI = 67%) in breast cancer xenograft model TN60–UNLP at 5 mg/kg dose
37 [130]	PPI	Peptide 9 exhibits a low IC_50_ value of 16 nM in the TR–FRET TEAD-binding assay. SPR dissociation constant (K_d_) of 25 nM X-ray structure of Peptide 9 in complex with TEAD4 reported.	NR
38 [131]	PPI	The apparent K_d_ of cyclo[E-LYLAYPAH-K] to N-terminal YAP1 was 1.75 μM and 0.68 µM for the WW domains of YAP1	NR
39 [132]	PPI	Isothermal titration calorimetry (ITC) showed that Fragment 1 binds to mTEAD4 with an affinity in the range of ~300 μM. Human TEAD2 X-ray structure (PDB code: 3L15) with analog Fragment 5 reported.	NR
40 [133,134]	PPI	In the EdU assay measuring the proliferation of SK-OV-3 and ES-2 cells, Peptide 17 inhibited proliferation by ~50% compared to the control.	Peptide 17 showed significant tumor inhibition in vivo (0.2 mg/kg, IP) in an SK-OV3-ip3-luc orthotopic mouse model (total flux ×10^6^ of ~15 versus ~45 for the control group)
41 [135,136]	PPI	SPR experiments measuring the binding of peptide TB1G2 to biotinylated TEAD immobilized on an SPR chip were conducted by means of single-cycle kinetic analysis and the K_d_ was determined to be 368 pM	NR
42 [137]	PPI	Fragment hit shows ΔT_m_ = 1.8 °C in the thermal shift assay with mTEAD4 Fragment hit occupies a site close to the YAP1-binding pocket on the TEAD surface	NR
43 [138]	PPI	cycDSβS[19–32] added with a TAT cell permeation sequence showed cytotoxic effects on esophageal cancer cell line EC109 with IC_50_ of 18.1 μM	NR
44 [139]	PPI	YAP1 TBD-derived cyclic peptide YSP-2 binds to TEAD with K_d_ binding affinity of 14 μM by FP assay.	NR
45 [140]	PPI	Stapled YAP1 α-helix peptide mutant sYAPm1 binds to TEAD with K_d_ affinity of 56 μM by SPR	NR
46 [141]	PPI	YAP1–TEAD AlphaLISA assay IC_50_ = 0.083 μM	NR
47 [142]	PPI	TEAD–GAL4 transactivation assay IC_50_ = 0.86 μM	NR
48 [143]	PPI	TEAD–GAL4 transactivation assay IC_50_ = 0.26 μM	NR
49 [144]	PPI	TEAD–GAL4 transactivation assay IC_50_ = 0.10 μM	NR
50 [145]	PPI	TEAD reporter luciferase activity inhibition (IC_50_ = 1.7 μM) observed in HEK293T cells treated with Compound 53 after 24 h post-transfection Compound 53 downregulated endogenous TEAD target genes CYR61, ANKRD1 and CTGF by ~50% using RTqPCR in MDA-MB-231 cells Cell viability of the MDA-MB-231 cells decreased using Compound 53 with CC_50_ = 6.9 μM	NR

The section below highlights several recent examples from Table 1 that have not been reviewed before, as well as emphasizes the molecules that have either entered the clinic or are positioned to do so in the near future.

### 3.1. MGH (Entry 6)

Wu et al. have previously disclosed the discovery and characterization of adamantyl-containing MGH–CP1 inhibitor of autopalmitoylation of endogenous TEAD in cells, with the allosteric mechanism and palmitate pocket binding subsequently confirmed by co-IP and the X-ray structure, respectively [146]. In a more recent patent application, the authors merged the adamantyl aniline portion of MGH–CP1, which extends deep into the lipophilic pocket, with the flufenamic acid structure to arrive at two key analogs: CP-58 and CP-55. In a cell proliferation assay, CP-55 is quite potent with IC_50_ values of 38 nM in 92.1 cells and 330 nM in Huh7 cells [98].

### 3.2. Basilea Pharmaceutica (Entry 7)

Basilea Pharmaceutica is a Swiss biopharmaceutical company that recently described in a patent application a series of 1.2,4-oxadiazol-5-one derivatives [99]. Structurally, these molecules show similarity with flufenamic acid [91], where the carboxylic acid group has been replaced with an oxadiazol-5-one warhead. These molecules show TEAD2 binding by thermal shift assay with a range of stabilizations from 0.4–18 °C and inhibit luciferase activity in the MCF7 TEAD reporter gene assay with a range of activities between 39 nM and 6 µM. Notably, Example 2 (MCF7 TEAD RGA IC_50_ = 41 nM, thermal shift assay hTEAD2 ΔTm = 10.6 °C, no PK data reported) is active in vivo in a 62-day murine NCI-H226 xenograft efficacy model by showing dose-dependent activity and tumor stasis at the highest dose of 250 mg/kg PO QD.

### 3.3. Genentech (Entries 8–11)

Researchers at Genentech have recently disclosed the full characterization of Compound 2 (GNE-9886) as a potent TEAD binder that blocks *S*-palmitoylation, as measured by Lipid FP assay [100]. Despite inhibiting palmitoylation, mechanistically, this molecule does not disrupt the YAP1–TEAD interaction, but rather is hypothesized to drive its reported in vivo efficacy (e.g., 78% TGI in the Detroit X1 562 xenograft model) by transforming TEAD into the dominant negative transcriptional repressor which blocks the TEAD interaction with chromatin. More recently, related analogs of GNE-9886 were patented where the noncovalent Cys interaction with the sulfonamide in Compound 2 was replaced with a covalent acrylamide warhead to yield representative Examples 5 and 53 [102]. While the affinity of these molecules for the TEAD palmitate pocket was retained, it is unclear from the presented data whether the switch to covalent binding has any impact on YAP1–TEAD complex disruption.

A novel, structurally distinct class of pan-TEAD binders was recently disclosed, with GNE-7883 highlighted as the lead reversible allosteric inhibitor of the YAP1–TEAD interaction [104]. The potent inhibition of pan-TEAD lipidation with GNE-7883 translated well to the disruption of YAP1 binding to each of the four TEAD isoforms by the YAP1 HTRF assay. GNE-7883 potently inhibited proliferation of the OVCAR-8 and NCI-H226 cells, with EC_50_ values of 115 nM and 333 nM, respectively. At 250 mg/kg (subcutaneous dosing), this molecule showed strong in vivo tumor growth inhibition of 102% in the mesothelioma NCIH226 xenograft mouse model, with a favorable tolerability profile based on the reported body weight change data relative to placebo. Overall, GNE-7883 represents an attractive molecule to further probe the Hippo pathway in cancer and the impact of allosteric inhibition of the YAP1–TEAD complex on efficacy across different indications and genetic backgrounds.

### 3.4. Ikena Oncology (Entries 12–13)

Ikena Oncology, a Boston-based biotechnological company, has recently published two patent applications disclosing their TEAD palmitoylation inhibitors. In the first patent application [106], a series of noncovalent aryl sulfonamide derivatives bearing a heterocycle (e.g., imidazole) and an amine (e.g., aniline or benzylamine) are described. These compounds show potent inhibition of TEAD reporter activity in MCF7 cells (EC_50_ range from <0.1 µM to >0.5 µM) and proliferation of H226 cells while maintaining selectivity against the Hippo WT H28 cell line (H226 EC_50_ range from <0.1 µM to >0.5 µM). Example I-27 in Entry 13 (MCF7 TEAD cell reporter assay EC_50_ < 100 nM, H226 cell proliferation assay EC_50_ < 100 nM and selectivity in the H28 cell proliferation assay with EC_50_ > 500 nM) is further exemplified with both pharmacokinetic and pharmacodynamic data. When administered at 10 mg/kg PO in BALB/c mice, I-27 reaches C_max_ = 1037 ng/mL and AUC_0-last_ = 2252 ng·h/mL. After 3-day treatment at 30 mg/kg PO in immunodeficient mice bearing NCI-H226 tumors, I-27 led to >50% downregulation of CTGF mRNA in the tumor, a known transcriptional target of YAP1–TEAD.

In the second patent application [105], a series of covalent derivatives with a variety of warheads is described (e.g., α-chloroketone, acrylamides, 3-bromodihydroisoxazole, 3-substituted dihydroisoxazole). Similar to the first patent application, compounds show potent inhibition of the TEAD reporter assay in MCF7 (EC_50_ range from <0.1 µM to >0.5 µM), with a select few also evaluated for inhibition of H226 proliferation and selectivity against the Hippo WT H28 cell line (H226 EC_50_ range from <0.1 µM up to >0.5 µM). Example I-186 is active in the MCF7 TEAD cell reporter assay with EC_50_ < 100 nM and inhibits H226 cell proliferation with EC_50_ < 100 nM while maintaining selectivity against H28 (EC50 >500 nM). PK/PD and efficacy data are reported for examples I-12 (Isomer 2) and I-186 (Isomer 1). Example I-12 (Isomer 2) administered at 10 mg/kg PO in BALB/c mice shows C_max_ = 23 ng/mL and AUC_0-last_ = 48 ng·h/mL, as well as > 50% downregulation of tumor CTGF mRNA upon 3 days of treatment at 50 mg/kg IP of mice bearing NCI-H226 tumors. Example I-186 (Isomer 1) administered at 10 mg/kg PO in BALB/c mice shows C_max_ = 110 ng/mL and AUC_0-last_ = 435 ng·h/mL, as well as >50% downregulation of tumor CTGF mRNA upon 3 days of treatment at 30 mg/kg PO of mice bearing NCI-H226 tumors. Both compounds were evaluated in the NCI-H226 tumor xenograft mouse model and showed promising antitumor activity after 28 days of treatment (e.g., 80% TGI for I-12 (Isomer 2) at 200 mg/kg PO QD dosing and 67% TGI for I-186 (Isomer 1) at 75 mg/kg PO QD dosing).

### 3.5. A*STAR (Entry 17)

While most reported palmitate pocket binders show inhibitory activity on TEAD palmitoylation and transcription, Hong and Probatti et al. recently reported quinolinol Q2 with palmitate-like TEAD stimulating activity [109]. Importantly Q2 has been shown to occupy the TEAD central pocket by SPR with a K_d_ = 2.6 µM and increase endogenous expression levels of TEAD target genes CTGF, CYR61 and ANKRD1 by RT-PCR. In vivo, quinolinol Q2 accelerates cutaneous wound healing in mice to as soon as day 7, which is consistent with the previously reported genetic experiments. This molecule further highlights the potential of modulating the Hippo pathway for wound healing and regenerative medicine that warrants further attention.

### 3.6. Vivace Therapeutics (Entries 18–24)

Vivace Therapeutics, a San Francisco-based biotechnological company, is one of the most active companies in the Hippo field with seven recent patent applications [110,111,112,113,114,115,116]; they also entered the clinic with their lead molecule VT3989 in early 2021. The Vivace patents can be characterized by two distinct chemical series. The first series derives from flufenamic acid [111,112,114,115,116] and consists of an aryl amine central core substituted with a five-membered heterocycle (e.g., imidazole, oxadiazole, tetrazole). The second series consists of a substituted bicyclic aromatic ring (e.g., naphthyl, quinoline), which contains an amide substitute and an aryl or an O-linked aryl group [110,113].

Within the first chemical series, compound VT-103 (Entry 18) and Compound 11 (Entry 21) [111,114] are derivatives of the representative Compound 23 (Entry 22) [115] where a carbonyl or a sulfone/sulfonamide group are introduced in the para-position of the aniline. Compounds 6 and 45 (Entry 23) [112] lack this third substitution, but the five-membered heterocycle is always a 1,3,4-oxadiazole. The reported cellular activities of these molecules in the reporter assay feature IC_50_ < 100 nM. In the second chemical series, the main difference between biaryl Compound 4 (Entry 20) [113] and VT-104 (Entry 18) [110] is the introduction of a flexible linker, mostly oxygen-based, between the aryl group and the bicyclic central core. As with prior compounds, the best reported activities in the cellular reporter assay feature IC_50_ < 100nM.

A series of key molecules included in the two abovementioned patent families are detailed in a recent paper by Vivace, which highlights the properties and characteristics of these TEAD inhibitors [33]. Among the compounds highlighted in the paper, VT-103 and VT-104 (Entry 18) are the most advanced examples, with both in vitro and in vivo data included. Based on the thermal shift and TEAD palmitoylation assay data, VT-103 is considered to be a TEAD1-selective binder, with low nanomolar IC_50_ in the proliferation assay using a panel of YAP1/TEAD-dependent cell lines. VT-103 has a favorable pharmacokinetics profile in mice with sufficient exposure for constant target engagement with daily oral dosing. VT-103 was evaluated in two malignant mesothelioma tumor xenograft mouse models with impressive outcomes, including (1) an NCI-H226 model with TGI = 106% at 3 mg/kg PO and (2) an NCI-H2373 model with TGI = 126% at 10 mg/kg PO.

VT-104 is part of the second family of bicyclic aromatics substituted with an amide functional group. Based on the thermal shift and TEAD palmitoylation assay data, VT-104 is a pan-TEAD binder, with low nanomolar IC_50_ values in proliferation assays using YAP1/TEAD-dependent cell lines. Crystal structure (PDB: 7CNL) of a close analog of VT-104 in the TEAD3 palmitoylation pocket confirms the noncovalent binding mode and palmitate pocket MOA of this derivative. VT-104 also exhibits a favorable pharmacokinetics profile with exposure sufficient for daily dosing in mice. In the NCI-H226 xenograft efficacy model, VT-104 is highly active with a TGI = 102% at 3 mg/kg PO. 

### 3.7. Astra Zeneca (Entry 28)

Astra Zeneca reported the design and characterization of Proteomimetic 7 (Tat–PEG_2_–4E), a stabilized ternary protein structure that disrupts the PPI between TEAD and its corepressor VGL4 [121]. Tat–PEG_2_–4E was derived from the VGL4 233-252 amino acid sequence and crosslinked via lactamization of residues E235 and K250. By SPR, 4E shows binding to hTEAD1 with a K_d_ = 0.7 µM. As a disruptor of the suppressive VGL4–TEAD complex, Tat–PEG_2_–4E significantly stimulates the expression of TEAD target genes CYR61, CTGF, ANKRD1 and SEPINE1 in human cardiomyocytes. In addition, it showed increased levels of YAP1 nuclear translocation and cell cycle activity in primary juvenile rat heart cells which is assumed to be required for cardiomyocyte proliferation. The cell cycle activity was stimulated by Tat–PEG_2_–4E to the same extent as positive controls SB203580 (p38MAPK inhibitor) and CHIR99021 (GSK3β inhibitor), thus highlighting the importance of peptidomimetic PPI inhibition of repressor complexes towards the activation of transcription factors.

### 3.8. University of Lille (Entry 50)

Compound 53 [145] is an optimized analog of the previously reported Hit 2 (Entry 26) [119] with an improvement in activity in the TEAD reporter assay; IC_50_ = 1.7 µM. Compound 53 inhibits the proliferation of MDA-MB-231 cells with CC_50_ = 6.9 µM and in the same model decreases TEAD target genes CYR61, ANKRD1 and CTGF by 50%. On the basis of thermal shift and molecular modeling tests, it has been proposed that Compound 53 is a classical and direct protein–protein interaction inhibitor of YAP1–TEAD. 

## 4. Clinical Trials Overview

The exciting progress in targeting the Hippo pathway described above has recently culminated in three molecules shown in Table 2 reaching the clinic in 2021. Amongst the various cancer indications where the Hippo pathway is deregulated, malignant mesothelioma is the primary indication selected in these clinical studies due to the high unmet medical need and the highest reported frequency of somatic mutations in NF2–Hippo, although other solid tumor indications will also be evaluated as discussed below.

Vivace Therapeutics is the sponsor of a phase I clinical study (NCT04665206) to evaluate the safety, tolerability, PK and biological activity of **VT3989**, a reported TEAD inhibitor in patients with refractory metastatic solid tumors, including refractory pleural malignant mesotheliomas. The chemical structure of **VT3989** has not been disclosed, but several patent applications have been published and reviewed above [110,111,112,113,114,115,116].

Ionis Pharmaceuticals is developing **ION537**, an antisense oligonucleotide (ASO)-targeting YAP1 mRNA which is currently undergoing a phase I trial in patients with molecularly selected advanced solid tumors [147,148]. **ION537** is administered by subcutaneous (SC) or intravenous (IV) injection, with the chemical structure recently presented at the AACR 2021 meeting as A_ks_A_ds_G_ds_T_ds_G_ds_T_ds_A_ds_T_ds_G_ds_T_ds_^m^G_ds_A_ks_G_es_A_ks_A_es_G_k_. **ION537** also corresponds to compound number 1198440 in a recent Ionis patent application [147]. Structurally, the backbone is modified with a phosphorothioate linker between each ribose for added stability, while a 2′–5′ constrained ethyl modification is introduced at positions 1, 12, 14 and 16 of the ASO for improved affinity, stability and tolerability. Additional modification of the ribose with a 2′ methoxyethyl (MOE) group is also introduced at positions 13 and 15. 

**ION537** inhibits cell proliferation in YAP1-activated head and neck tumors (SCC25 GI_50_ = 80 nM; BIC56 GI_50_ = 80 nM). In addition, **ION537** inhibits YAP1 mRNA expression in several tumor xenografts models: (1) in the human hepatocellular carcinoma SNU449 xenograft tumor model, Yap1 mRNA is decreased by 51% after five days of treatment with 50 mg/kg **ION537** twice weekly, (2) in the human epidermoid carcinoma A-431 xenograft tumor model, Yap1 mRNA is decreased in tumors by 32% after five days of treatment with 25 mg/kg **ION537** daily, (3) in the human squamous cell carcinoma CAL27 xenograft tumor model, Yap1 mRNA is decreased in tumors by 67% after five days of treatment with 15 mg/kg **ION537** daily, and (4) in the CAL33 human squamous cell carcinoma xenograft tumor model, Yap1 mRNA is decreased by 88% in tumors after five days of treatment with 50 mg/kg **ION537** twice weekly. In the same experiment, the weight of the tumor decreased by 74% compared to the vehicle after 34 days of treatment.

The latest arrival in the clinic, Novartis, has initiated a phase I clinical study with a molecule **IAG933**. The purpose of that study is to characterize the safety and tolerability of **IAG933** in patients with mesothelioma, NF2/LATS1/LATS2-mutated tumors and tumors with functional YAP1/TAZ fusions, as well as to identify the maximum tolerated dose. The structure, the binding partner, and the mechanism of action of **IAG933** have not been disclosed yet, although a new patent application has recently been disclosed by Novartis, at the time of publication of this review [149].

## 5. Concluding Remarks

The past decade and a half of Hippo/YAP1 research has uncovered countless connections to human diseases ranging from Ebola [150] to oncology and even applications in laboratory-grown meat [151]. This wide array of biological functions has sparked a plethora of research targeting the pathway, and in particular the YAP1–TEAD interaction. We have now entered a new phase of investigation, where the safety and relevance of YAP1–TEAD in driving human disease will be tested clinically, in particular for oncology and tumors such as malignant mesotheliomas. In addition, the disclosure of structures of selective and potent compounds that disrupt the YAP1–TEAD association could allow for the probing of more biological questions that cannot be easily answered with genetic tools, particularly, which tumor indications may respond to the YAP1–TEAD dissociation. This line of investigation could lead to an expansion of potential indications outside of malignant mesothelioma tumors.

## Figures and Tables

**Table 2:** Recently initiated clinical trials to inhibit TEAD-driven transcription in cancers.

Compound (Ref.)	Structure	Phase	Disease Indication	Sponsor (Trial Number)
**VT3989**	Not disclosed	I	Malignant pleural mesothelioma with mutations of NF2 Solid tumor patients with mutations of NF2	Vivace Therapeurics (NCT04665206)
	
**ION537** [147,148]	A_ks_A_ds_G_ds_T_ds_G_ds_T_ds_A_ds_T_ds_G_ds_T_ds_^m^G_ds_A_ks_G_es_A_ks_A_es_G_k_	I		
			Advanced solid tumors	Ionis Pharmaceuticals (NCT04659096)
**IAG933**	Not disclosed	I	Malignant pleural mesothelioma Solid tumors with loss-of-function NF2/LATS1/LATS2 genetic alterations Solid tumors with functional YAP1/TAZ fusions	Novartis (NCT04857372)

## Data Availability

Not applicable.

## Abbreviations

AACR	American Association for Cancer Research
ABT	1-Aminobenzotriazole
ALK	Anaplastic lymphoma kinase
ANKRD1	Ankyrin repeat domain 1
AXL	AXL receptor tyrosine kinase
BCLXL	B cell lymphoma-extra large
BMF	Bcl2-modifying factor
CETSA	Cellular thermal shift assay
Co-IP	Complex immunoprecipitation
CTGF	Connective tissue growth factor
CXCL5	C–X–C motif chemokine 5
CYR61	Cysteine-rich angiogenic inducer 61
DSS	Dextran sodium sulfate
EdU	Ethynyl deoxyuridine
EGFR	Epidermal growth factor receptor
EML4	Echinoderm microtubule-associated protein-like 4
EMT	Epithelial mesenchymal transition
FOS	Fos proto-oncogene, AP-1 transcription factor subunit
FP	Fluorescence polarization
HER3	Human epidermal growth factor receptor 3
HTRF	Homogeneous time-resolved fluorescence
IP	Intraperitoneal
ITC	Isothermal titration calorimetry
KRAS	Kirsten rat sarcoma virus
LATS	Large tumor suppressor kinase
MAPK	Mitogen-activated protein kinase
MDSC	Myeloid-derived suppressor cell
MEK	MAPK/ERK kinase
mKRAS	Mutant KRAS
MMP7	Matrix metalloproteinase-7
MOA	Mechanism of action
MOE	2′-O-methoxyethyl
MST	Mammalian sterile 20-like kinase
NF2	Neurofibromin 2
NFAT1	Nuclear factor of activated T cells, cytoplasmic 2
NR	Not reported
NSCLC	Non-small-cell lung carcinoma
PDB	Protein Data Bank
PD-L1	Programmed death-ligand 1
PO	Per os (oral dosing)
PPI	Protein–protein interaction
QD	Quaque die (once a day dosing)
RGA	Reporter gene assay
ROS1	Proto-oncogene tyrosine-protein kinase
SLUG	Snail family transcriptional repressor 2
SPR	Surface plasmon resonance
SRC	Proto-oncogene tyrosine-protein kinase Src
TAZ	WW domain-containing transcription regulator protein 1
TBK1	TANK-binding kinase 1
TCGA	The Cancer Genome Atlas
TEAD	TEA domain family member
TGI	Tumor growth inhibition
TME	Tumor microenvironment
TREG	Regulatory T cells
VGL4	Vestigial like family member 4
YAP1	Yes-associated protein 1
YBD	YAP-binding domain
